# *In silico* Structure–Based Investigation of Key Residues of Insecticidal Activity of Sip1Aa Protein

**DOI:** 10.3389/fmicb.2020.00984

**Published:** 2020-05-29

**Authors:** Jing Wang, Ming-Yue Ding, Jian Wang, Rong-Mei Liu, Hai-Tao Li, Ji-Guo Gao

**Affiliations:** ^1^College of Life Sciences, Northeast Agricultural University, Harbin, China; ^2^State Key Laboratory for Biology of Plant Diseases and Insect Pests, Institute of Plant Protection, Chinese Academy of Agricultural Sciences, Beijing, China

**Keywords:** *Bacillus thuringiensis*, Sip1Aa, site-directed mutation, structure analysis, *Colaphellus bowringi* Baly

## Abstract

*Colaphellus bowringi* Baly mainly damages cruciferous vegetables, leading to huge economic losses. The secretory insecticidal protein (Sip) of *Bacillus thuringiensis* (Bt) has high insecticidal activity against *C. bowringi* Baly. The tertiary structure of Sip1Aa protein was analyzed by homologous modeling and other bioinformatics methods to predict the conserved domain of Sip1Aa protein. Acidic and basic amino acids in the conserved domain were selected, and alanine was used to replace these amino acids by site-directed mutation. The difference between the insecticidal activities of mutant protein and Sip1Aa protein was analyzed. The insecticidal activities of H99A, K109A, K128A, and E130A against *C. bowringi* Baly were significantly increased, among which that of K128A was the most obviously changed, and the LC_50_ value was decreased by about 10 times compared with that of Sip1Aa protein. The LC_50_ value of mutant E130A was 0.286 μg/mL, which was about six times less than that of Sip1Aa. K128 and E130 were both in the β9–β10 loop. The toxicity of D290A, H242A, and H303A to *C. bowringi* Baly was significantly reduced, and their LC_50_ value increased by about six, eight, and three times compared with that of Sip1Aa protein, respectively. This study showed that acidic and basic amino acid residues played a certain role in the toxicity of Sip1Aa protein, and the loss of side chains in key residues had a significant impact on the insecticidal activity of the protein. This study provides the theoretical basis for revealing the relationship between the structure and function of Sip1Aa protein and also provides a new method for the subsequent study of *sip* gene.

## Introduction

*Bacillus thuringiensis* (Bt) is a gram-positive Bacterium that has been widely used in the control of agricultural and forestry pests such as *Lepidoptera*, *Diptera*, and *Coleoptera* due to its high specificity to target pests and non-target biosecurity (Schnepf et al., 1998). It is the most successful biological insecticide for major agricultural pests at present (Xu et al., 2018). Insecticidal protein are mainly Insecticidal crystal proteins (Cry) (Donovan et al., 2001, 2006; Milne et al., 2008), Vegetative insecticidal proteins (Vips) (Estruch et al., 1996), and Secreted insecticidal proteins (Sip) (Donovan et al., 2006). Among them, Cry and Vip have been studied deeply, and many kinds have been discovered, but little is known about Sip.

Currently, there are several research reports on Sip. Donovan studied the insecticidal activity of Bt strains on *Coleoptera* and found that the culture supernatant of some strains has insecticidal activity on the larvae of Colorado potato beetle (CPB) at the lethal dose 50 (LD_50_) of 0.12 (0.09∼0.15) μg/mL. The researchers further cloned the new gene, which contains 1104 bases and encodes 367 amino acids. The gene was named Sip1A (Donovan et al., 2006). The similarity between Sip1A and the mosquitocidal protein Mtx3 is 46% (Promdonkoy et al., 2003). In 2012, the *sip* gene containing 1038 bp and encoding 345 amino acid sequences was cloned and identified from another Bt strain QZL26 in our laboratory, but the insecticidal activity was not reported (Liu et al., 2012). Murawska et al. (2013) applied genome sequencing technology and showed that strain IS5056 contained the *sip* gene, but no further study was conducted. In addition, our laboratory cloned a *sip* gene that contains 1188 bp and encodes 395 amino acid sequences from Bt strain DQ89 in 2015, which showed high toxicity to *Colaphellus bowringi* Baly, with LC_50_ of 1.542 mug/mL (Jinbo et al., 2015).

In addition, Sha Junxue cloned a novel *sip* gene that contains 1095 bp and encodes 364 amino acids from Bt strain QZL38, which was 95% similar to the nucleic acid sequences of the known *sip1A* gene, and named it as *sip1Ab*. Then, she constructed a truncated mutant, amplified and expressed the *sip1Ab* coding sequence that contained 1005 bp after the first 90 bp signal peptide was removed, and encoded 334 amino acids, and she renamed it as Sip1Aa, whose size was 37.6 kDa. The assay results of the insecticidal activity showed that the Lethal Concentration 50 (LC_50_) of the *C. bowringi* Baly was 1.051 μg/mL (Sha et al., 2018).

Site-directed mutagenesis is widely used in the study of the function of a particular amino acid residue in a protein. Alanine substitution is widely used to analyze the biological functions of amino acid residues in protein molecules. Since the side chain of alanine is relatively small, many residues can be substituted by alanine without affecting the overall structure of the protein molecule (Cunningham and Wells, 1989; Howlader et al., 2010). Hu et al. (2018) predicted that the amino acid residues of Cry1Ac (Met341, Asn442, and Ser486) and CR7–CR11 (Asp32, Arg101, and Arg127) were hotspot residues involved in the interaction of toxin receptor complexes through computer-assisted alanine scanning mutations. Ward et al. (1988) mutated acidic and basic amino acids into alanine to explore its role in polar phospholipid group interaction. Chi et al. (2018) and Luo et al. (2018) applied site-directed mutagenesis in the study of key sites of the insecticidal activity of Vip3Aa11 protein. Yang et al. (2014) applied site-directed mutagenesis in the study of improving the activity and stability of the enzyme. Ming et al. (2017) applied site-directed mutagenesis in the study of the insecticidal mechanism of Vip3Aa protein. Wang et al. (2012) predicted the tertiary structure model of Cry5Ba by homologous modeling and studied the structural and functional importance of asparagine residues in the block 3 sequence of the Cry5Ba subfamily by alanine scanning mutation. In the study of the insecticidal mechanism of Cry protein, it was found that proteolytic activation may be an important limitation for Cry toxin. After proteolytic cleavage, it was relatively easier to access the epithelial cells of the core toxin than that of protoxin. The specific association between the hydrolyzed toxin and the brush border membrane vesicle (BBMV) is stronger than that between the original toxin and BBMV (Deist et al., 2014). CRY toxin breaks down the midgut cells of larvae after forming small pores (Pacheco et al., 2018; Xu et al., 2018). In the study of the insecticidal mechanism of Vip protein, it was found that Vip3Aa protein was activated by midgut proteases, which passed through the peritrophic membrane and were bound to the specific proteins in the midgut cells of the apical membrane, leading to the formation of pores and ultimately the death of insects (Ming et al., 2017). However, information about the insecticidal mechanism of Sip protein has not been reported so far.

In the past studies of Sip, the structural information was often overlooked. Although the structural information of Sip protein is still unknown, the structural analysis of the Sip protein can be theoretically accomplished by homology modeling. In this study, the bioinformatics research method was used to predict the tertiary structure of Sip1Aa and to guide the site-directed mutagenesis test. The conserved domain of Sip1Aa protein was predicted, and the alanine scanning mutations were performed on the acidic and basic amino acid residues in the conserved domain. 18 mutant proteins H99A, K109A, K128A, E130A, D134A, D136A, D141A, K145A, K193A, H242A, H259A, D290A, R292A, D299A, H303A, H318A, and D328A were successfully constructed. We selected *C. bowringi* Baly, an insect of *Coleoptera*, that is widely distributed in the northeast part of China. The insecticidal activity of these mutant proteins was determined, and the insecticidal activity of some mutant proteins showed a difference from that of wild-type protein. This study provides the theoretical basis for explaining the relationship between the structure and function of Sip1Aa and provides the genetic resources for the prevention and control of *Coleoptera* (Aronson and Shai, 2001; Hu and Aroian, 2012; Fuente-Salcido et al., 2013), providing guidance for the study of the structure and activity mechanism of Sip1Aa protein.

## Materials and Methods

### Computer-Aided Modeling of the Three-Dimensional of Sip1Aa Protein

The Sip1Aa protein (undisclosed) was discovered in our laboratory. According to Chi et al. (2018), the amino acid sequence of Sip1Aa was submitted to the automated protein structure homology-modeling server (Phyre^2^)^1^ and run in intensive mode to generate the multi-template modeling (Kelley et al., 2015). The resulting 3D model was submitted to the ModRefiner server^2^ for optimization, and then the model was evaluated by RAMPAGE^3^. The Ramachandran plot was used to confirm the stability and reliability of the simulated conformations. The DEEP VIEW SWISS—PDB VIEWER software from the EXPASY server^4^ was used to visualize and analyze the atomic structure of the model. Finally, PyMOL from the Molecular Graphics System was used to produce the figures.

### Bacterial Strains and Plasmids

*Escherichia*. *coli* (*E. coli*) JM109 and BL21(DE3) were used as the host strains for the cloning and expression studies. Vectors derived from pET-28a(+) were used for the production of recombinant protein. The Bt strain QZL38 harboring the wild *sip1Aa* gene was maintained in the laboratory.

### Primers and the Construction of Site-Directed Mutagenesis

pET28a-*sip1Aa* (pET28a-carrying *sip1Aa* gene) was stored in our laboratory, cloned, and methylated in the *E. coli* strain of JM109 as a template. The eighteen primers used for the construction of mutagenesis are shown in Table 1. Reverse PCR amplification was conducted on the closed circular DNA, and 1 μL *Dpn*I enzyme was added into 50-μL PCR product; after mixing well, they were incubated at 37°C for 2 h. The linearized product was recyclized with Mut Express II Fast Mutagenesis Kit V2 (Vazyme Biotech, Nanjing, China), then the cyclic products were transformed into the *E. coli* BL21 strain. Positive transformants were screened using primers Sip-F/Sip-R, and the mutant plasmids were sequenced. After sequencing by Comate Bioscience Co., Ltd, the mutants were, respectively, named as H99A, K109A, K128A, E130A, D134A, D136A, D141A, K145A, K193A, H242A, H248A, H259A, D290A, R292A, D299A, H303A, H318A, and D328A.

**TABLE 1:** PCR primers used for amplification.

Primer	Primer sequence 5′–3′
Sip-F	ccgaattcgagctccgtcgacATGGCAGAAACCAAGTCGCCAA
Sip-R	gtggtggtggtggtgctcgagATTTCCACTTAAAATCTTTGTTTGA
H99A-F	CAATgcTCAAACAAATAGATTTATATCCTGGTTTAA
H99A-R	CTATTTGTTTGAgcATTGTTATTTTGATCTTTAAATATCCAGAA
K109A-F	CCTGGTTTgcAGATAATCTTGCTAGTTCGAAGGGG
K109A-R	GATTATCTgcAAACCAGGATATAAATCTATTTGTTTGAT
K128A-F	GGGCTTAgcAATAGAAGCATTAAATGATATGGATGTAAC
K128A-R	GCTTCTATTgcTAAGCCCATTTGTTCTGCTATACTG
E130A-F	GGCTTAAAAATAGcAGCATTAAATGATATGGATGTAACAAATA
E130A-R	GCTgCTATTTTTAAGCCCATTTGTTCTGCTAT
D134A-F	GCATTAAATGcTATGGATGTAACAAATATTGATTATACATCTAA
D134A-R	TCCATAgCATTTAATGCTTCTATTTTTAAGCCC
D136A-F	TGATATGGcTGTAACAAATATTGATTATACATCTAAAACAGG
D136A-R	TTGTTACAgCCATATCATTTAATGCTTCTATTTTTAAGC
D141A-F	GcTTATACATCTAAAACAGGTGATACCATATATAA
D141A-R	GTTTTAGATGTATAAgCAATATTTGTTACATCCATATCATTTAATG
K145A-F	TCTgcAACAGGTGATACCATATATAATGGAATTT
K145A-R	GGTATCACCTGTTgcAGATGTATAATCAATATTTGTTACATCCATATC
K193A-F	AGGATTTgcAGTTGCTGCTAAGGGAGTAGTTGC
K193A-R	GCAGCAACTgcAAATCCTAATTGTAACCCATTTGTTACT
H242A-F	TATCCCCAGGAgcTAAAGCAGTGGTGAAACATGATTTG
H242A-R	CTTTAgcTCCTGGGGATAATGTAACTTCTTGAG
H248A-F	GCAGTGGTGAAAgcTGATTTGAGAAAAATGGTGTATTTTG
H248A-R	ATCAgcTTTCACCACTGCTTTATGTCCTGGGGA
H259A-F	GGTGTATTTTGGGACTgcTGATTTAAAGGGTGATTTAAAAGTAGGT
H259A-R	gcAGTCCCAAAATACACCATTTTTCTCAAATCA
D290A-F	GATTTATCTGcTATTCGTAAAACAATGATTGAAATTGA
D290A-R	CGAATAgCAGATAAATCAATTGATCTATAATTTGGAT
R292A-F	CTGATATTgcTAAAACAATGATTGAAATTGATAAATGG
R292A-R	TTGTTTTAgcAATATCAGATAAATCAATTGATCTATAATTTGG
D299A-F	TGcTAAATGGAATCATGTAAATACCATTGACT
D299A-R	CATGATTCCATTTAgCAATTTCAATCATTGTTTTACGAATATC
H303A-F	TGGAATgcTGTAAATACCATTGACTTTTATCAATTAGTT
H303A-R	GGTATTTACAgcATTCCATTTATCAATTTCAATCATTGT
H318A-F	GTTGGAGTTAAAAATgcTATAAAAAATGGTGATACTTTATAT ATAGATACCC
H318A-R	AgcATTTTTAACTCCAACTAATTGATAAAAGTCA
D328A-F	ATATATAGcTACCCCGGCCGAATTTACATTTA
D328A-R	CCGGGGTAgCTATATATAAAGTATCACCATTTTTTATATGATTTT

### Expression and Extraction of Protein of Sip1Aa and the Mutants in *E. coli*

Recombinant *E. coli* BL21(DE3) strains harboring the *sip1Aa* gene or mutant genes were pre-cultured overnight at 37°C and 220 rpm in 5 mL of the LB medium containing 100 μg/mL kanamycin. The culture was transferred to 100 mL of LB medium. When *OD*_600_ reached 0.6, isopropyl-β-D-thiogalactopyranoside (IPTG) was added to achieve the final concentration of 1.5 mM. The culture continued to grow for additionally 14 h at 16°C and 160 rpm. The final culture was centrifuged at 8000g for 5 min at 4°C. The supernatant was discarded and the cells were resuspended in 30 mL of pre-chilled PBS buffer (20 mM sodium phosphate, 0.5 M NaCl, pH 7.4), and the process was repeated for two times, then recentrifuged at 8000g for 5 min at 4°C, the pellet was resuspended with 5 mL PBS buffer (pH 7.4). The bacteria were broken by lysozyme and ultrasonic vibration in an ice-water mixture (Ampl 80%, pulse on 3 s, pulse off 3 s, 10 min), then centrifuged at 12,000g for 15 min at 4°C in order to remove the insoluble material. Finally, the supernatant was filtered through a 0.22-μm filter, and the pellet was resuspended in PBS buffer (pH 7.4) and the suspension was collected. All the collections were analyzed by SDS-PAGE electrophoresis, and the estimation of Sip1Aa protein and mutant protein concentration was performed with BSA standards and the software of ImageJ.

### Purification of Mutant Protein

Since the pET28a vector was labeled with histidine, a nickel column was used for purification. According to the method in the protein purification kit (ComWin Biotech, Beijing, China), add the deionized water of 5 column volumes to the filled column to rinse the ethanol, then balance the column with binding buffer (20 mmol/L Na_3_PO_4_, 0.5 mol/L NaCl, 40 mmol/L imidazole) of 10 column volumes. At the end of equilibration, add 5 mL of soluble protein. Rinse the column with the binding buffer of 15 column volumes to remove the impurities. The purified protein was collected by elution with an appropriate amount of elution buffer (20 mmol/L Na_3_PO_4_, 0.5 mol/L NaCl, 500 mmol/L imidazole) and verified by SDS-PAGE. After elution, the column was washed with deionized water of 10 column volumes, and then the column was balanced with 20% ethanol of 3 column volumes. The column was sealed and stored at 2∼8°C.

### Insects and Bioassays

The standard *C. bowringi* Baly used in this study was donated by the Institute of Plant Protection (IPP), Chinese Academy of Agricultural Sciences (CAAS). Analysis of the toxicity in *C. bowringi* Baly was conducted on second instar larvae with fresh cabbage using the leaf-dip bioassay (Tabashnik et al., 1993), performed in triplicate using different concentrations of crude-extracted mutant protein and the Sip1Aa protein as well as the insecticidal protein solution of PBS buffer as the control. An empty plasmid was used as the negative control. For each concentration and control, 16 s instar larvae of *C. bowringi* Baly were used. The number of dead insects was recorded, and the insect mortality was calculated after 2 days of larvae exposure at 27°C, 55 ± 5% RH, and a 14/10-h light/dark cycle. The corrected mortality rate of mutant protein to insects was calculated according to the number of dead larvae in the control group. In addition, after purification, the soluble protein was diluted into six concentration gradients for the measurement of insecticidal activity; the LC_50_ value was measured with POLO-PC software. Each bioassay was repeated in triplicate.

## Results

### Construction of Protein Three-Dimensional Models

**FIGURE 1:**
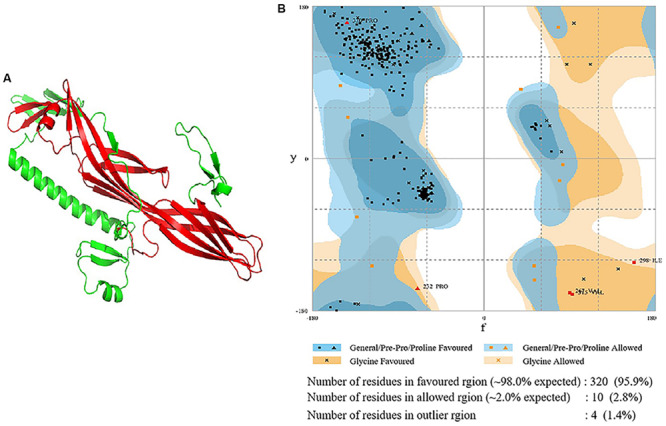
Construction and analysis of a three-dimensional structure model of Sip1Aa protein. **(A)** Predicted the three-dimensional structure model of Sip1Aa protein. **(B)** The Ramachandran plot for evaluating the model.

The Sip1Aa protein was modeled with multiple templates, and the PDB IDs of the templates were d1uyja, c4rhzA, c3d42B, c4znoB, c4pkmA, c1w3gA, d3c0na2, c2ztbB, c3c0mB, and d1tzoa, respectively. The homologous region of d1uyja in the first place covers 25% of the Sip1Aa protein. The model was optimized with ModRefiner and pulled closer to its original state based on hydrogen bonding, skeleton topology, and side-chain positioning. From the structural model of Sip1Aa protein, it was seen that in the tertiary structure of Sip1Aa protein, most of them were β-sheets and only a few were α-helices; the location of the conservative domain is shown in red in Figure 1A. The evaluation result of the model is shown in Figure 1B. The Ramachandran plot showed the distribution of Φ-Ψ dihedral angles in the main chain of the model. The Φ-Ψ angle of the 95.9% amino acid residues in the Sip1Aa protein model was in the core region, and only 1.4% of the residues were in non-permissive regions, and the amino acid residues in the non-permissive were not within the scope of study. It showed that the dihedral angle of amino acid residues in the model was reasonable and that the model was dependable.

### Expression and Extraction of Mutant Protein in *E. coli*

**FIGURE 2:**
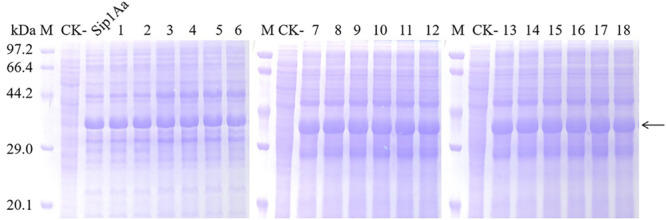
Expression of mutant soluble protein in *E. coli.* M: Protein marker (low), CK-: pET28a, Sip1Aa: Expression of wild-type Sip1Aa protein in *E. coli*; 1: H99A, 2: K109A, 3: K128A, 4: E130A, 5: D134A, 6: D136A, 7: D141A, 8: K145A, 9: K193A, 10: H242A, 11: H248A, 12: H259A, 13: D290A, 14: R292A, 15: D299A, 16: H303A, 17: H318A, and 18: D328A.

Expression from recombinant strains was induced by IPTG. The expression of Sip1Aa protein in *E. coli* was used as the positive control, and the expression of pET28a in *E. coli* was used as the negative control. SDS-PAGE analysis is shown in Figure 2. The results showed that all mutants were successfully expressed in *E. coli*, and the expressed product contained a fusion protein of 37.6 kDa, which was consistent with Sip1Aa protein.

### Bioassay

**FIGURE 3:**
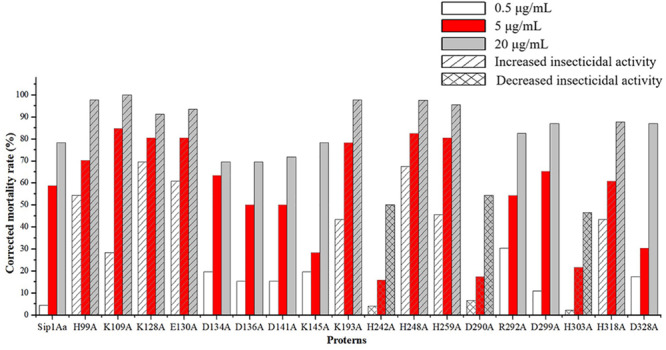
Qualitative bioassay results of mutant protein and Sip1Aa protein against *Colaphellus bowringi* Baly.

The expression from recombinant strains was induced by IPTG. In the qualitative bioassays for the insecticidal activity of mutant protein, second-instar *C. bowringi* Baly were used as tested insects, and in that of the protein expressed in *E. coli*, pET28a was used as the negative control. The protein concentrations for bioassay were, respectively, 0.5, 5, and 20 μg/mL. Forty-eight insects were tested for each protein, and the death of insects was counted 48 h later. The experiment was repeated for three times. The test results are shown in Figure 3. The lethal rates of the mutant protein H99A, K109A, K128A, E130A, K193A, H248A, H259A, and H318A to *C. bowringi* Baly were higher than that of Sip1Aa protein, and the insecticidal activities of mutant protein H242A, D290A, and H303A against *C. bowringi* Baly were lower than that of Sip1Aa protein. There was no significant difference between the lethal rates of the mutant protein D134A, D136A, D141A, K145A, R292A, D299A, and D328A and the Sip1Aa protein to *C. bowringi* Baly.

**FIGURE 4:**
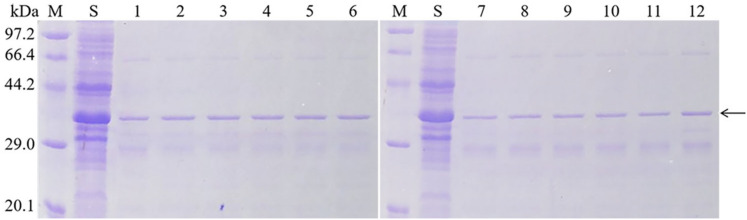
Purification of mutant soluble protein. M: Protein Marker (Low), Sip1Aa: Sip1Aa crude protein, 1: Purified Sip1Aa protein, 2: H99A, 3:K109A, 4: K128A, 5: E130A, 6: K193A, 7: H242A, 8: H248A, 9: H259A, 10: D290A, 11: H303A and 12: H318A.

Based on the results of qualitative bioassay, H99A, K109A, K128A, E130A, K193A, H242A, H248A, H259A, D290A, H303A, and H318A were purified (Figure 4) and subjected to quantitative bioassay against *C. bowringi* Baly. PBS buffer was used as negative control. The concentration gradients for the insecticidal activity of *C. bowringi* Baly were 0.1, 1, 5, 10, 20, and 50 μg/mL. Bioassay results are given in Table 2.

**TABLE 2:** Quantitative bioassay of mutant proteins against *Colaphellus bowringi* Baly.

Proteins	LC_50_ (μg/mL)	95% confidence interval	Slope ± SD
Sip1Aa	1.683	1.135–2.409	1 ± 0.1
H99A	0.665	0.186–1.493	0.7 ± 0.1
K109A	0.481	0.283–0.734	1 ± 0.1
K128A	0.18	0.033–0.457	0.5 ± 0.1
E130A	0.286	0.094–0.586	0.6 ± 0.1
K193A	1.62	0.895–2.706	0.7 ± 0.1
H242A	13.186	9.292–19.933	1 ± 0.1
H248A	1.069	0.294–2.579	0.9 ± 0.1
H259A	1.221	0.834–1.720	1 ± 0.1
D290A	9.308	5.349–18.855	0.6 ± 0.1
H303A	4.728	2.636–9.016	0.6 ± 0.1
H318A	1.235	0.717–1.954	0.8 ± 0.1

The insecticidal activities of H248A, H259A, and H318A against *C. bowringi* Baly were slightly better than that of Sip1Aa protein, but there was no significant difference. The insecticidal activities of the mutants H99A, K109A, K128A, and E130A against *C. bowringi* Baly were significantly better than that of Sip1Aa protein, and the LC_50_ values were reduced by about 3 times, 4 times, 10 times, and 6 times. The insecticidal activities of H242A, D290A, and H303A against *C. bowringi* Baly were significantly lower than that of Sip1Aa protein, and the LC_50_ values were increased by about 8 times, 6 times, and 3 times, respectively. There was no significant difference in the insecticidal activity of mutant K193A against *C. bowringi* Baly compared with that of Sip1Aa protein.

### Structural Analysis of Mutation Sites

**FIGURE 5:**
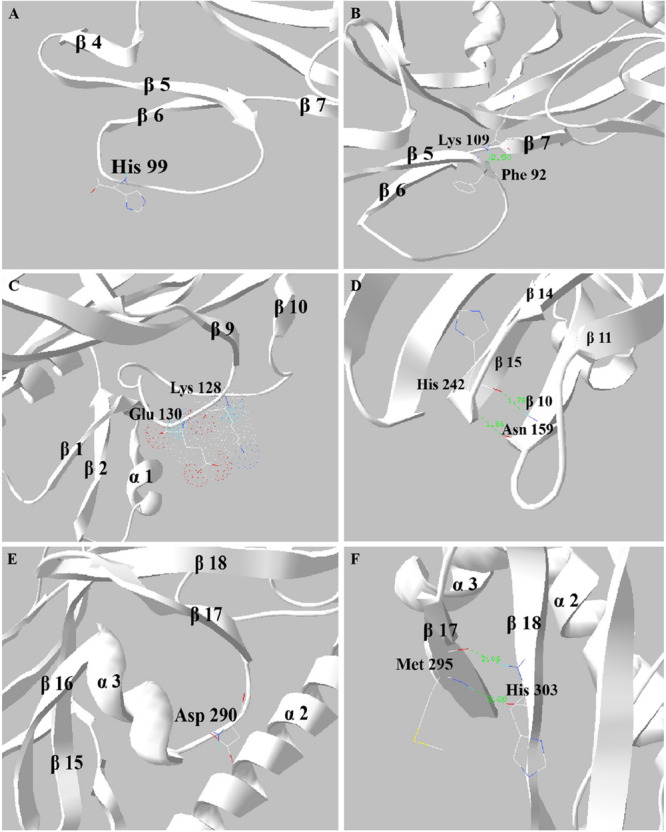
Structural analysis of mutant sites. **(A–F)** The structural information His99, Lys109, Lys128 and Glu130, His242, Asp290, and His303.

Structural analysis of the H99, K109, K128, E130, H242, D290, and H303 sites of Sip1Aa protein was performed with the software of PDB Viewer. The 99th histidine of Sip1Aa protein was in the middle of the β5–β6 loop, as is shown in Figure 5A. After H99 was replaced by alanine, the side chain was removed and the steric hindrance was reduced, resulting in the mutant’s increased insecticidal activity against *C. bowringi* Baly. K109 located on the β6–β7 loop formed intramolecular hydrogen bonds with phenylalanine at position 92, as is shown in Figure 5B. According to hydrophobic analysis, K109 was located on the outside of the protein molecule and might be involved in the binding of Sip1Aa protein to the receptor. When the hydrogen bond is broken, K109 may bind more easily to the ligand, thereby increasing the insecticidal activity of the protein. Lysine at position 128 of Sip1Aa protein and glutamate at position 130 were located in the loop at positions β9–β10, both of which have unstable side chains, as is shown in Figure 5C. The insecticidal activity of K128A and E130A mutants to *C. bowringi* Baly has been improved, possibly because the electron cloud becomes smaller after the mutation of K128 or E130 into alanine, which smoothes the loop structure on the surface and makes it easier to complete the binding with insect receptors. Aspartic acid at position 290 of Sip1Aa protein is located on the alpha 3-beta 17 loop, as is shown in Figure 5E. When D290 was replaced by alanine, the insecticidal activity of the protein decreased by about six times, suggesting that D290 plays an important role in the toxic effect of the protein. The 242th histidine was located on β15 of Sip1Aa protein and formed two hydrogen bonds with asparagine at position 159 on β10, and the distance values between the hydrogen bonds were 1.78 Å and 1.86 Å, as is shown in Figure 5D. The 303rd histidine was located on β18 of Sip1Aa protein and formed two hydrogen bonds with methionine at position 295 on β17, and the distance values between the hydrogen bonds were 2.0 and 2.06 Å, as is shown in Figure 5F. The interaction between H242 and N159 and that between H303 and M295 maintained the stability of the structure. H242 and H303 were mutated in alanine, which lost two stable intramolecular hydrogen bonds and might lead to an antibiotic change in the steric structure and a decrease in the insecticidal activity of the protein against *C. bowringi* Baly.

## Discussion

*Colaphellus bowringi* Baly (Chrysomelidae: Coleoptera) is widely distributed in northeastern China. It is fond of cruciferous vegetables, causing a large reduction in the production of cruciferous vegetables and resulting in huge economic losses. *B. thuringiensis* has been used worldwide for genetically modified plants or spraying pesticides to control agricultural pests (Pacheco et al., 2009; Pardo-Lopez et al., 2009; Rodriguez-Almazan et al., 2009). The Bt Sip protein has a high toxic effect on the *C. bowringi* Baly; however, the mechanism of its insecticidal activity is still unclear. As previously studied, Sip1A protein has high insecticidal activity in Colorado potato beetle (Donovan et al., 2006). Therefore, Sip protein may have some insecticidal activities against other Coleoptera insects, which needs further study.

In order to explore the key sites of insecticidal activity of Bt Sip1Aa protein, the three-dimensional structure model of Bt Sip1Aa protein was successfully predicted by the bioinformatics research method. However, the structure information of mutants cannot be obtained accurately by homology modeling because the spatial structure of the template is followed first in the process of model building. Therefore, it is necessary to construct mutant protein for functional verification. A portion of the acidic and basic amino acids were selected in the conserved domain for alanine scanning mutations and 18 mutants of H99A, K109A, K128A, E130A, D134A, D136A, D141A, K145A, K193A, H242A, H248A, H259A, D290A, R292A, D299A, H303A, H318A, and D328A were successfully constructed, all of which were able to form inclusion bodies and successfully express 37.6 kDa soluble protein, indicating that these amino acid residues were replaced by alanine without destroying the advanced structure of the protein.

Hydrophobicity analysis of Sip1Aa protein showed that lysine 109 was located on the surface of the protein molecule, which might be related to the binding of the intestinal receptor to the target insect. When K109 was replaced by alanine, a hydrogen bond between K109 and F92 was also cleaved, which may enhance the ability of the mutant protein to bind to the target cells, but further experiments such as ligand binding are needed to prove this subsequently. When K128 was replaced by alanine, the insecticidal activity against *C. bowringi* Baly was significantly increased, and its LC_50_ value was reduced by about 10 times compared with Sip1Aa. Among these mutant proteins, the change in toxin activity was most pronounced when Lys128 was substituted with alanine. The conformation changed when K128 was replaced by alanine; it might enhance the ability to bind and lyse target cells *in vitro*. However, more experiments are needed to test this hypothesis. When E130 was replaced by alanine, the LC_50_ value of mutant E130A was 0.286 μg/mL, which was about six times less than that of Sip1Aa. K128 and E130 were both in the β9–β10 loop, and after the two amino acid residues were replaced by alanine, the insecticidal activity of the *C. bowringi* Baly was significantly increased. It is presumed that this loop plays an important role in the tertiary structure of the Sip1Aa protein.

Three mutations involving the substitution of acidic amino acid (D290A) and basic amino acids (H242A and H303A) resulted in a significant decrease in the insecticidal activity against *C. bowringi* Baly, indicating that these residues are located in the position of critical toxicity in the tertiary structure. The mutations in conserved amino acid residues can affect the insecticidal activity of protein. For example, the insecticidal activity of H242A against *C. bowringi* Baly was about eight times lower than that of Sip1Aa, which indicates that this amino acid site may be a key site in the core active region of Sip1Aa protein. In addition, the interaction of H242 with N159 stabilizes the spatial configuration of the region. To clarify the function of H242 and the reasons for the decrease in insecticidal activity, further experiments are needed. The data obtained from the analysis of mutant protein indicated that the charged residues affecting the insecticidal activity were distributed throughout the conserved domain. However, the change in D290 did result in considerable loss of insecticidal activity, indicating that it had a critical position in the overall tertiary conformation of the molecule. The significant decrease in the insecticidal activity of D290A might be due to the abnormal conformation caused by the loss of charge at this position, which affected the normal function of the core region of the toxin. In order to verify the demand for acidic amino acid at this position, D290 can be changed to E290 so as to investigate whether the insecticidal activity of the mutant protein can be restored to the level of that of the wild-type protein.

Without the exact structure information of Sip1Aa protein, it is always a difficult problem to explore the relationship between its structure and function. In this study, site-directed mutagenesis was combined with bioinformatics to explore the effects of specific amino acid residues on protein function, which laid a foundation for studying the insecticidal mechanism of Sip1Aa protein. At the same time, highly active mutants were obtained, which provided a new method for expanding the Bt insecticidal spectrum and subsequent research on the *sip* gene. However, the biological activity of the *sip* gene to Coleoptera insects needs further screening and analysis, which lays a solid foundation for subsequent research. This also provides materials for the study of genetically modified and engineered bacteria.

## Data Availability Statement

All datasets generated for this study are included in the article/supplementary material.

## Author Contributions

JinW, H-TL, and J-GG contributed to the conception and the design of experiments. JinW, JiaW, and M-YD performed the experiments. R-ML looked up part of the literature. JinW and H-TL conceived the study and analyzed the results. All authors read and approved the final manuscript.

## Conflict of Interest

The authors declare that the research was conducted in the absence of any commercial or financial relationships that could be construed as a potential conflict of interest.

## Footnotes

1 http://www.sbg.bio.ic.ac.uk/phyre2/html/page.cgi?id=index

2 http://zhanglab.ccmb.med.umich.Edu/ModRefiner

3 http://mordred.bioc.cam.ac.uk/~rapper/rampage.php

4 https://www.expasy.org/
